# Alcohol’s Negative Emotional Side: The Role of Stress Neurobiology in Alcohol Use Disorder

**DOI:** 10.35946/arcr.v42.1.12

**Published:** 2022-10-27

**Authors:** Rajita Sinha

**Affiliations:** Yale University School of Medicine, New Haven, Connecticut

**Keywords:** alcohol, distress, craving, relapse, negative emotions, neural activity, glucocorticoids

## Abstract

This article is part of a Festschrift commemorating the 50th anniversary of the National Institute on Alcohol Abuse and Alcoholism (NIAAA). Established in 1970, first as part of the National Institute of Mental Health and later as an independent institute of the National Institutes of Health, NIAAA today is the world’s largest funding agency for alcohol research. In addition to its own intramural research program, NIAAA supports the entire spectrum of innovative basic, translational, and clinical research to advance the diagnosis, prevention, and treatment of alcohol use disorder and alcohol-related problems. To celebrate the anniversary, NIAAA hosted a 2-day symposium, “Alcohol Across the Lifespan: 50 Years of Evidence-Based Diagnosis, Prevention, and Treatment Research,” devoted to key topics within the field of alcohol research. This article is based on Dr. Sinha’s presentation at the event. NIAAA Director George F. Koob, Ph.D., serves as editor of the Festschrift.

The word “alcohol” often conjures up positive feelings and associations with fun, socializing, relaxing, and partying. Yet there is another side to drinking alcohol, especially with risky, hazardous levels of consumption. This side is associated with distress and may include anxiety, loneliness, pain, and depressive symptoms.^1^ This has been labeled the “dark side,” or “negative emotional, stress side,” of alcohol intake.^2^ These two paradoxical, dialectically opposing alcohol experiences map onto the biphasic drug effects of alcohol, with alcohol being both a stimulant and a depressant drug. They also represent a shift from positive to negative situations that may drive alcohol intake, especially as alcohol intake increases from low or moderate “social” levels of drinking to binge, heavy, and chronic consumption. The National Institute on Alcohol Abuse and Alcoholism (NIAAA) defines drinking in moderation as an intake of two drinks or less per day for men and one drink or less per day for women. Binge drinking is generally defined as five or more drinks per occasion for men and four or more drinks per occasion for women. Heavy drinking is generally defined as more than four drinks per day or more than 14 drinks per week for men and as more than three drinks per day or more than seven drinks per week for women.^3^

One aspect of the research the author has conducted with the support of NIAAA, and which is the topic of this article, has focused on identifying the physiological and neural effects, as well as the subjective and cognitive effects, of binge and chronic alcohol use. This research also has explored the factors that influence these effects and investigated whether these effects can be reversed or normalized to allow for recovery from any of the long-term changes that occur with binge and chronic alcohol misuse.

The worldwide coronavirus (COVID-19) pandemic is a chronic, ongoing stressor. Research has shown that alcohol consumption has increased significantly during this period, especially among individuals who regularly binge drink or drink heavily.^4^,^5^ While onsite alcohol sales were down as businesses closed, e-commerce profits increased more than 30% during the COVID-19 pandemic.^4^,^5^ Who is most susceptible to increased drinking episodes during COVID-19–related stress? This question highlights the need to understand the well-known bidirectional relationship between stress or trauma and alcohol intake, and why those with binge and chronic alcohol use are most vulnerable to increased alcohol use under high levels of stress and with traumatic exposure.

This article reviews human research investigating neurobiological and psychological changes related to alcohol misuse that are associated with greater distress and stress-related alcohol craving and their role in predicting risk of binge drinking, relapse, and impact on treatment outcomes. The author presents the effects of stress and trauma on brain stress responses and their associations with resilient coping and describes the impact of binge and chronic alcohol use on brain and peripheral stress responses and their role in promoting alcohol craving and relapse risk. Specific clinical and biobehavioral markers of both risk of developing alcohol use disorder (AUD) and relapse are also reviewed. Finally, the article discusses recent findings on treatments that focus on reversing stress and craving disruptions related to chronic alcohol misuse to improve treatment outcomes.

## Alcohol and Stress—Shift From Positive to Negative Effects

It is well known that one or two standard alcoholic drinks have a stimulating and physiologically arousing effect; for example, heart rate increases acutely, and blood pressure changes have been documented. These responses are part of the autonomic nervous system readouts that occur with alcohol intake, but also are observed in challenging situations such as when faced with acute stressful life events.^6^,^7^ The arousing response to alcohol is associated with a sense of feeling energized and stimulated as well as increases in sociability.^6^ With increasing levels of alcohol intake in one sitting, however, alcohol also stimulates the hypothalamic-pituitary-adrenal (HPA) axis, and increases in cortisol are observed.^8^,^9^ Alcohol also activates brain emotion and stress pathways, including the amygdala, under emotional arousing and stressful states.^10^,^11^ In addition, acute alcohol use stimulates the brain cortico-striatal pathways involved in reward, motivation, and goal-directed behaviors. These include the ventral and dorsal striatum, the orbitofrontal cortex (OFC), and the ventromedial prefrontal cortex (VmPFC).^10^–^13^ The emotion/stress pathway and the reward/motivation pathways closely interact, and such interactions are involved in emotional cue-related drinking motivation.^11^,^12^

Binge and hazardous alcohol drinking patterns are associated with well-documented changes both in the brain stress and emotion regions, such as the amygdala,^8^,^12^ and in associated brain networks, including the ventral and dorsal striatum as well as the OFC, VmPFC, and dorsolateral prefrontal cortex.^9^,^12^,^14^,^15^ These brain changes are associated with blunted autonomic and cortisol responses to stress and to acute alcohol intake,^6^,^8^ as well as with increases in negative emotional and stress responses and greater alcohol craving.^6^,^9^,^14^–^17^ Together, these changes are part of the psychobiological adaptations in humans that occur with increasing patterns of binge and hazardous alcohol intake.

### Stress, Alcohol Craving, and Binge Alcohol Intake

Acute stress exposure stimulates the autonomic, endocrine, and brain emotion and motivation regions that process and regulate negative emotion and distress responses, and it also activates stress coping.^6^,^12^,^18^ Additionally, acute stress exposure increases physiological arousal, including cortisol responses, and activates brain stress pathways involved in emotional arousal, emotional learning, and memory. This activation occurs via circuits involving the hypothalamus, amygdala, hippocampus, insula, and prefrontal regions, including the OFC, VmPFC, and inferior frontal cortices. Also activated is the premotor supplementary motor area, which is involved in behavioral intent, response selection, and action.^6^,^18^,^19^ Previous studies reported that there are dynamic time-dependent changes in the cortico-striatal regions involving the ventral and dorsal striatum and the VmPFC during stress versus non–stress conditions; these changes were associated with active, goal-directed stress coping.^18^ Additionally, greater dynamic responses in these brain stress-reward pathways were associated with lower daily numbers of alcoholic drinks consumed, lower reports of emotional conflicts, and lower emotional eating, whereas blunted ventral striatum and VmPFC responses during stress were associated with greater reports of binge drinking, emotion dysregulation, and emotional eating.^18^ Based on these findings, the dynamic neural responses in the striatum and VmPFC are thought to document neurophysiological flexibility during stress, and their associations with behavioral coping suggest that this circuit is part of the resilient stress-coping pathway involved in behavioral control and self-regulation of stress, emotions, and reward impulses.^6^,^18^

These adaptations to alcohol also vary by sex, as fundamental differences between men and women exist in brain organization, structure, and functional networks^20^ as well as in the responses of brain stress, emotion, and reward regions^21^ and in patients with cocaine use disorder.^22^ Moreover, sex differences in the responses to stress and to alcohol-related stimuli have been documented in people who drink moderately. Unlike in animal studies, males in human studies show greater adrenocorticotropic hormone (ACTH) and cortisol responses to stress,^23^ whereas females show higher autonomic physiologic arousal to stress; a greater response to stress cues in the amygdala, insula, OFC, and VmPFC; and greater VmPFC response to alcohol cues.^24^–^28^ This suggests that the psychological and biological responses to alcohol and to stress vary by sex and that although men and women report similar levels of alcohol motivation when matched for recent drinking history, the psychological and neurobiological pathways that facilitate alcohol use are different for men and women who drink moderately.

**Figure 1:**
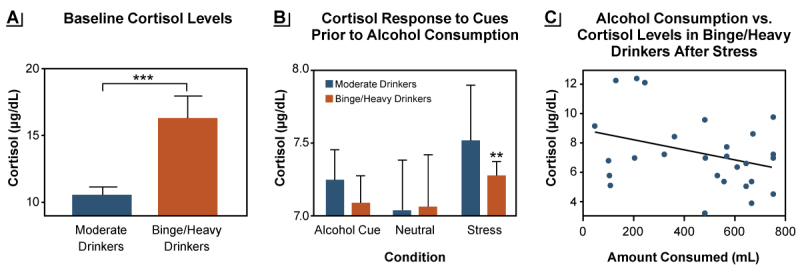
Baseline cortisol levels and responses to stress differ between moderate drinkers and binge/heavy drinkers (A) Fasting morning plasma levels of cortisol (μg/dL) were higher in binge/heavy drinkers (orange bars) compared to moderate drinkers (blue bars) (***p < .001). (B) Cortisol responses to stress and alcohol cues, but not to neutral cues, were blunted in binge/heavy drinkers compared with moderate drinkers (**p < .01). (C) In binge/heavy drinkers, the behavioral motivation for alcohol use as reflected in the amount of alcohol consumed post stress in an ad lib drinking task was greater in individuals with a more blunted cortisol response to stress (r^2^ = .11, p = .0022). *Source:* Adapted with permission from Blaine et al. (2019).^8^

Regardless of sex, repeated escalated alcohol use induces changes in both peripheral and brain stress systems.^2^,^12^,^16^ Higher binge levels of alcohol use increase basal cortisol levels and blunt the peripheral stress responses; these changes also predict greater craving and behavioral motivation for alcohol use in people who binge drink or drink heavily (see Figure 1).^8^,^9^ Additionally, changes in the amygdala responses to emotional cues and ventral striatal responses to alcohol have been reported with higher binge levels of alcohol use.^14^,^29^ Along with these neural changes, increased salience of alcohol and greater alcohol craving levels have been observed in response to stress as well as in response to alcohol and to alcohol cues, which then promote increased alcohol intake and escalation to risky drinking.^8^,^15^,^17^ These brain stress system, physiologic, and behavioral effects of binge drinking history need to be further examined by sex to better understand the recent data on greater escalation of binge drinking in women compared to men.^30^

### Effects of Stress and Trauma on Brain Pathways and AUD Risk

Stress and trauma are associated with greater levels of risky alcohol intake as well as greater severity of AUD.^19^ Numerous different types of traumatic stress and life events as well some temperament and individual-level variables relate to risk of binge drinking and developing AUD (see Table 1). Exposure to repeated stress and trauma also contributes to changes in the brain and body’s responses to stress and emotions as well as to changes in alcohol motivation and adaptive coping responses.

**Figure 2:**
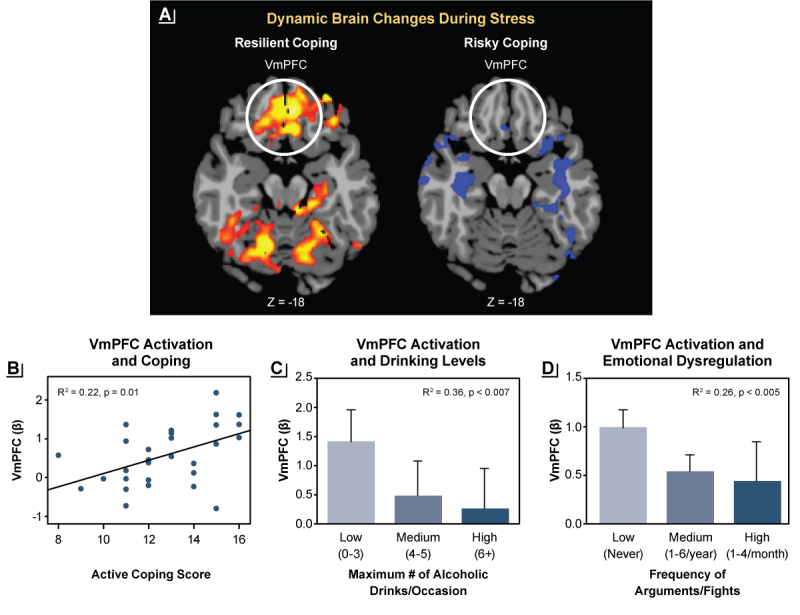
Associations between brain stress responses and resilient coping (A) Dynamic activation in the ventromedial prefrontal cortex (VmPFC) during stress challenge (represented by red and yellow) was a sign of resilient coping, whereas a lack of dynamic changes in the VmPFC during stress, suggesting inability to mobilize during stress, was a sign of risky coping. (B) Greater dynamic activation of the VmPFC was associated with greater self-reported active coping. (C) Lack of dynamic activation of the VmPFC was more pronounced in binge drinkers. (D) Greater emotional dysregulation (measured by greater frequency of arguments or fights) also was associated with less dynamic activation of the VmPFC. *Source:* Adapted with permission from Sinha et al. (2016).^18^

Greater levels of cumulative adversity, stressful life events, and trauma are associated with lower brain volume and greater negative emotion and subjective stress responses. They also are associated with dysregulated neural and peripheral physiological responses to stress and to alcohol cues in the brain regions involved in stress, emotion, reward regulation, and self-control, including the OFC, VmPFC, supplementary motor area, amygdala, insula, and striatum.^31^–^33^ Furthermore, altered or blunted ACTH and cortisol and autonomic responses to stress and to alcohol and drug cues are observed with greater trauma or stress.^19^,^33^ These stress- and trauma-related brain and peripheral alterations co-occur alongside emotional and behavioral dysregulation and higher alcohol motivation. As a result, people with more risky drinking exposed to stress or trauma are at greater risk of emotion dysregulation as evidenced by more arguments, fights, emotional eating, and higher maximum drinks consumed per occasion (see Figure 2).^18^,^34^

Several interacting brain networks are activated during stress, including those involved in emotion experiences (e.g., amygdala, insula), emotional memory (e.g., amygdala, hippocampus), reward and motivation regions (e.g., ventral and dorsal striatum), and goal-directed behavior (e.g., OFC, VmPFC).^13^,^18^,^19^,^21^,^29^ These regions form networks and patterns of activation that enable emotional and motivational coping, and both stress and alcohol directly act on these networks to influence active coping, motivation, and flexible control of behavior, such as exercising self-control with drinking. The accumulating evidence shows that stress and trauma exposure alter these emotional and motivational responses involved in adaptive stress coping, such that people become more vulnerable to craving and consuming higher levels of alcohol, which increases risk of hazardous and risky drinking.

The research described above resulted in the development of a model explaining the role of glucocorticoids in drinking behavior on the basis of changes in peripheral cortisol levels and responses across the full spectrum of alcohol consumption levels.^8^ At baseline, people who binge drink or drink heavily have higher cortisol levels than those who drink moderately (see Figure 1A), indicating a shift in HPA axis functioning. This also suggests possible changes in brain glucocorticoid pathways in humans that may increase risk of hazardous drinking. As stated earlier, alcohol consumption stimulates cortisol release; however, in response to either stress or alcohol exposure, the increase in cortisol is lower in people who binge drink or drink heavily than in those who drink moderately. Thus, when given one standard alcoholic drink, those drinking at binge levels do not feel its effects as robustly as do people who drink moderately.^8^,^9^ As cortisol is critical for survival, humans have well-preserved neurobehavioral signals with the brain stress system pathways^12^ that seek to enhance cortisol release in response to stress. In people with blunted cortisol responses due to heavy drinking, this mechanism may signal greater motivation for alcohol to increase alcohol-related cortisol responses.^9^ Thus, there is a neurophysiologic drive to enhance wanting alcohol in order to increase cortisol and HPA axis functioning in people who drink heavily. This disruption in alcohol-related cortisol signaling and the need to drive the homeostatic HPA axis rhythm back to functional levels may be one component of the enhanced motivation for alcohol in those who drink alcohol at binge and heavy levels. This conceptual model suggests that normalizing the brain and body’s stress and motivational coping responses may reduce risk of hazardous drinking. Researchers are seeking to develop and evaluate novel strategies to achieve this normalization and to reduce the risk of heavy drinking.

## Effects of Stress and Alcohol Cues in AUD

Researchers also have investigated the role of stress biology and stress responses in people with AUD. Chronic heavy drinking or binge drinking increases the risk of disrupted alcohol-related autonomic and HPA axis responses as described in previous sections. These disruptions contribute to clinical symptoms associated with the negative emotional side of AUD,^15^ such as increased levels of anxiety, negative mood, sleep difficulties, emotional reactivity, and impulsivity, along with high levels of craving for alcohol.^1^,^35^ Furthermore, these disruptions increase the risk of relapse and heavy drinking during treatment and posttreatment, thereby jeopardizing long-term recovery.^6^,^36^,^37^ Alcohol relapse refers to return to heavy drinking (at binge levels) after any period of abstinence, whereas treatment failure refers to maintaining or returning to binge and hazardous drinking levels during or after treatment.^3^ These observations have led researchers to investigate which factors contribute to early risk of dropout and recovery failure during treatment.

A series of studies assessed brain and body responses as well as cognitive, emotional, and motivational responses to both stress and alcohol cues in a laboratory study of human participants with AUD who were entering treatment and control participants without AUD. The analyses also included structural and functional magnetic resonance imaging as well as real-world daily assessment of stress and motivational responses using smartphones. These analyses using multiple approaches across different samples of individuals with AUD found that stress exposure increased alcohol craving. This response was accompanied by higher emotional, mood, and anxiety symptoms and lower ability to regulate emotions and control alcohol cravings.^36^,^37^ Furthermore, the biological stress response was significantly disrupted during the early recovery period. Thus, individuals in early recovery exhibited a higher basal heart rate and higher free cortisol levels, but lower levels of endogenous bound cortisol. Additionally, these individuals did not show a significant normal response to stress or alcohol challenge.^6^,^37^ Thus, the biological responses that support emotion and mood regulation are disrupted during this early recovery phase, and the greater these levels of dysfunction, the higher the risk of relapse or heavy drinking. Notably, sex differences in these biological responses have been reported, where women with AUD showed a more blunted ACTH and cortisol level than men with AUD; however, women had much higher basal norepinephrine levels, which in turn affected their response to stress and to alcohol cues.^26^,^38^

Another series of experiments examined brain correlates of later alcohol relapse and treatment failure. These analyses found that the volume of gray matter cells in the medial prefrontal brain regions—which are involved in regulating emotions, reward, and actions—was lower among individuals entering treatment compared with healthy control participants.^39^ Also, individuals with the lowest gray matter volume in the medial prefrontal brain region tended to be most likely to relapse and not do well in treatment.^39^ Analyses assessing the function of these brain regions during experimental exposure to stress and to alcohol cues (compared to neutral cues) detected disrupted, hyperactive VmPFC responses to neutral relaxing cues, but blunted, hypoactive VmPFC responses to stress and cue exposure. These observations suggest that the brain pathways that help regulate emotions and desires showed dysfunction and that the greater the VmPFC disruption, the higher the risk of alcohol relapse and heavy drinking.^40^,^41^

The studies described above have led to the characterization of a risk profile to identify individuals who are most vulnerable for alcohol relapse and heavy drinking during treatment. Thus, risk was determined by specific clinical measures—such as alcohol craving and withdrawal,^42^,^43^ mood, anxiety, and sleep difficulties—and biological markers^37^ as well as by additional moderating factors, including childhood maltreatment (see Table 2).^44^ Furthermore, this research supported the conceptualization that the effects of binge drinking and chronic alcohol use on stress biology occur along a continuum, with higher levels of alcohol intake associated with more significant chronic stress pathophysiology, which in turn contributes to greater risk of alcohol relapse and treatment failure.^35^

## AUD Treatments Targeting Stress, Craving, and Loss of Control of Alcohol Intake

Critical basic science and translational work by Koob and colleagues^45^ had focused on stress pathophysiology to develop novel therapeutics for AUD. Similarly, the findings described above motivated additional research to evaluate whether reversal of the chronic alcohol-related disruptions in stress psychobiology that are associated with increased alcohol craving and relapse risk could improve treatment and treatment outcomes for individuals most vulnerable to alcohol-related stress pathophysiology. Previous research by Arnsten had shown that noradrenergic agents such as guanfacine and prazosin could rescue the prefrontal cortex from the toxic effects of high uncontrollable stress.^46^ Because the effects of chronic alcohol exposure are similar to those of high chronic stress, it seemed plausible that pharmacologic targets that reduce prefrontal norepinephrine and the toxic effects of stress-related damage also could be of benefit in improving the stress and craving-related pathology associated with AUD. Studies to test these hypotheses have shown positive results. Guanfacine, an alpha-2 adrenergic agonist that reduces brain norepinephrine in the prefrontal cortex, improved prefrontal functioning and reduced alcohol and drug craving.^47^,^48^ Furthermore, guanfacine had some sex-specific effects, with greater benefits in women than in men.^49^,^50^

Similarly, prazosin—an alpha-1-adrenergic antagonist that had been shown to improve working memory and prefrontal functioning during stress^46^ as well as withdrawal-related drinking in laboratory animals^51^—reduced stress-related craving and stress dysfunction in AUD.^52^,^53^ Based on these findings, an NIAAA-supported, 12-week proof-of-concept, double-blind, placebo-controlled, randomized trial of prazosin versus placebo (16 mg/day, three times a day dosing, titrated over 2 weeks) was conducted with 100 individuals with AUD. The study found that alcohol withdrawal symptoms were a moderating factor impacting prazosin efficacy in improving drinking outcomes over 12 weeks; that is, prazosin treatment benefit was determined by the presence of alcohol withdrawal symptoms at treatment entry. Thus, individuals with more severe alcohol withdrawal symptoms at treatment initiation experienced greater reductions in heavy drinking days and drinks per occasion during the 12-week treatment period.^54^ In addition, prazosin reduced alcohol craving, anxiety, and negative mood compared with placebo in participants with high alcohol withdrawal symptoms, but had no impact in those with no or low levels of alcohol withdrawal symptoms. Finally, prazosin appeared to reverse VmPFC and dorsal striatal dysfunction, improving medial prefrontal response to stress and reducing dorsal striatal response to alcohol cues in participants treated with prazosin compared with those receiving placebo.^55^ These findings support further development of prazosin in the treatment of severe AUD. However, they also underscore the need to pursue further research to identify behavioral and pharmacologic strategies to prevent and treat chronic alcohol effects on stress pathophysiology in AUD.

## Conclusions

This article summarizes research by the author’s group demonstrating that binge, heavy, and chronic drinking leads to adaptations in brain, biological, and psychological stress responses. These adaptations are associated with alcohol’s negative emotional aspects, as evidenced by greater alcohol craving, higher alcohol withdrawal, greater negative mood and anxiety symptoms, as well as sleep difficulties that are commonly reported by individuals with AUD entering treatment. These changes occur in brain stress, reward, and motivation pathways that represent the stress pathophysiology of AUD. This stress pathophysiology directly targets brain circuits that underlie people’s ability to cope with stress and day-to-day challenges and are involved in jeopardizing recovery from AUD.

This research also has identified various clinical and biobehavioral markers that are associated with relapse and treatment failure and has allowed for identification of individuals who may be at greatest risk of treatment failure. Additionally, identification of these markers has led to research seeking to develop new strategies to target and reverse the stress pathophysiology of AUD to optimize interventions for AUD. Current and future work is focused on developing and testing specific treatments that can target this particular stress pathophysiology and help individuals who are most vulnerable to jeopardizing their recovery in the early phase of AUD treatment.

## Figures and Tables

**Table 1:** Types of Adverse Life Events, Trauma, Chronic Stressors, and Individual-Level Variables Predictive of Addiction Risk

Adverse Life Events	Childhood and Life Trauma	Chronic Stressors	Stressful Internal States
Loss of parent Parental divorce and conflict Isolation and abandonment Single-parent family structure Forced to live apart from parents Loss of child by death or removal Unfaithfulness of significant other Loss of home to natural disaster Death of significant other or close family member	Physical neglect Physical abuse by parent, caretaker, family member, spouse, or significant other Emotional abuse and neglect Sexual abuse Rape Victim of gun shooting or other violent acts Observing violent victimization	Being overwhelmed Unable to manage life problems Difficulties with job, living situation Financial problems Interpersonal conflicts, loneliness Unfulfilled desires Problems with children Illness of loved ones Negative emotionality Poor behavioral control Poor emotional control	Hunger or food deprivation Food insecurity Extreme thirst Sleep deprivation or insomnia Extreme hypothermia or hyperthermia Excessive drug use Drug withdrawal states Chronic illness

*Source:* Included with permission from Milivojevic & Sinha (2018).^37^

**Table 2:** Markers and Moderators Associated With Relapse to Alcohol Use and Treatment Failure in Alcohol Use Disorder (AUD)

Clinical and Biological Markers	Moderating Factors
Increased levels of alcohol craving High early physical, sexual, emotional abuse and trauma history High basal beat-by-beat heart rate and blunted autonomic response to stress and cues Altered bound and free fasting morning cortisol levels, and adrenal sensitivity Blunted and hypoactive cortisol response to stress Lower medial prefrontal gray matter volumes in magnetic resonance imaging Blunted medial prefrontal cortex response to stress and alcohol cues Hyperactive striatal responses to alcohol cues	AUD severity, including life span factors of early or late AUD; acute withdrawal symptoms, including anxiety, sleep, and negative mood; alcohol abstinence days Early physical, sexual, and emotional abuse and lifetime traumas; chronic stress; and trauma-related pathophysiology Sex differences and gender-related comorbid psychopathology and medical conditions Genetic and pharmacogenomic effects
